# miR-27a Downregulation Promotes Cutaneous Squamous Cell Carcinoma Progression via Targeting EGFR

**DOI:** 10.3389/fonc.2019.01565

**Published:** 2020-01-21

**Authors:** Yinghui Wang, Xuyi Deng, Yu Dai, Xinli Niu, Meijuan Zhou

**Affiliations:** ^1^Jiangmen Central Hospital, Affiliated Jiangmen Hospital of Sun Yat-sen University, Jiangmen, China; ^2^Department of Radiation Medicine, Guangdong Provincial Key Laboratory of Tropical Disease Research, School of Public Health, Southern Medical University, Guangzhou, China; ^3^Department of Dermatology, Nanfang Hospital, Southern Medical University, Guangzhou, China

**Keywords:** miR-27a, cutaneous squamous cell carcinoma, proliferation, metastasis, EGFR, UVB

## Abstract

Cutaneous squamous cell carcinoma (cSCC) is the second common malignant cancer around the worldwide and is etiologically linked to ultraviolet radiation. miRNAs play an important role in the initiation and progression of cancers. However, the functions of miRNAs in cSCC remain to be elucidated. Here, we screened and identified miR-27a as a consistently downregulated miRNA after UVB irradiation in HaCaT cells. It was found that miR-27a expression was significantly decreased in cSCC cells and tissues. *in vitro* and *in vivo* experiments showed that miR-27a inhibited cell proliferation and invasion of cSCC cells. Mechanistically, EGFR was identified to be directly targeted by miR-27a and miR-27a suppressed the phosphorylation of EGFR and its downstream NF-κB signaling pathway. Overall, these findings suggest that downregulation of miR-27a promotes tumor growth and metastasis via targeting EGFR and its downstream NF-κB signaling pathway, reminding that miR-27a plays a vital role in the progression of cSCC and could be a new therapeutic target.

## Introduction

cSCC is the second most common malignant cancer after basal cell carcinoma worldwide (1). The estimated total number of cSCC for 2012 has been increased by 100% as compared with 1992 (2), due to the increase exposure of ultraviolet from solar radiation and artificial sources. Although basal cell carcinoma and cSCC are both derived from epidermis, cSCC is highly invasive and can metastasize to distant organs (3). As the mechanism of cSCC tumorigenesis is very complex and poorly understood, local therapy, including targeting therapy is still deficient. In the majority of cSCC patients, tumors are excised by surgery conduction, which will result in the functional impairments and physical abnormities for skin tissues. Therefore, the mechanism of cSCC tumorigenesis is urgently further exploration and new strategies needed to be developed to reduce relapse and minimize facial defects.

MicroRNAs (miRNA) are a large family of small endogenous non-coding RNAs comprising 18–22 nucleotides, which directly bind to the 3′UTR region of its target genes through complete or incomplete complementary pairing (4). miRNAs participate in many biological processes, which include cell differentiation, cell survival, apoptosis. Extensive studies have demonstrated that miRNAs play an important role in tumor initiation, progression, and metastasis of various cancers (5, 6) and have emerged as promising therapeutic targets or tools for cancer treatment (7). Recent studies have shown that differential expressed miRNAs in cSCC compared with normal epidermis are associated with tumor initiation and progression (8–10). miR-27a is located on chromosome 19 and aberrant expressed in several types of cancers, including breast cancer (11), osteosarcoma (12), renal cell cancer (13). However, the role of miR-27a in the progression of cSCC has not been elucidated. Here, as ultraviolet radiation is the major risk for cSCC and cumulative sun exposure has a strong dose-response association with cSCC, we selected miR-27a, which is consistently downregulated in response to UVB radiation and aimed to investigate its function in the progression of cSCC, which would be a promising therapeutic target for cSCC treatment.

## Materials and Methods

### Cell Lines and Tissue Samples

cSCC lines HSC-1 and HSC-5 (HonSun Biological Co. Ltd., Shanghai, China) and immortalized human keratinocyte cell line HaCaT (CellCook Biotech Co. Ltd., Guangzhou, China) were cultured in Dulbecco's modified eagle medium (DMEM, Life Technologies) containing 10% fetal bovine serum (Invitrogen) and 100 μg/ml streptomycin and 100 units/ml penicillin in 37°C incubator with 5% CO_2_ and a mild atmosphere. CSCC samples were collected from patients who have been diagnosed by expert pathologists from January 2014 to August 2016 in the departments of dermatology, pathology, and oncology at Nanfang Hospital, affiliated to Southern Medical University. This study was approved by the Institutional Review Board of Nanfang Hospital affiliated to Southern Medical University and written informed consent provided from all patients for the use of surgical samples.

### UVB Irradiation

UVB exposure to HaCaT cells was performed as described previously (14). In brief, culture medium was removed and PBS was used to wash cells twice. PBS was replaced in a minimal volume and HaCaT cells were exposed to 30 mJ/cm^2^ of UVB. After irradiation, PBS was removed and the conditioned cultured medium was added back. HaCaT cells were incubated at 37°C and harvested at different time points. BALB/c mice (female, 4–6 weeks old, *n* = 6 per group) were shaved 24 h before UVB radiation. All animals received UVB exposure every other day at 300 mJ/cm^2^ (1/2 MED, minimum erythema dose) and mice skins were collected for further analysis after 4 weeks.

### Reverse Transcription and qPCR

Total RNA isolation was performed by using TRIzol (Life technologies) according to the manufacturer's instructions. Reverse transcription was performed by Mir-X miRNA First-Strand Synthesis Kit (Takara) and the expression of miRNA was measured using Taqman Mixture (CWBio, Shanghai, China). The data were normalized to U6 snRNA. PrimeScript RT Reagent Kit (Takara) was used to generate cDNAs and mRNA analysis were performed by UltraSYBR Mixture (CWBio, Beijing, China). GADPH was used as normalization. All qPCR reactions were performed on a LightCycler 96 Detection System (Roche). The primers are listed in Supplementary Material.

### Western Blot

The total protein of cells was extracted on ice by cell lysis buffer (Beyotime, Shanghai, China) mixed with protease inhibitor cocktail. BCA quantification kit (Beyotime, Shanghai, China) was used to determine protein concentration. Lysates were separated by SDS polyacrylamide gel electrophoresis. Proteins were blotted onto PVDF membranes (Millipore). These membranes were incubated with primer antibodies overnight at 4°C and then secondary HRP-conjugated antibodies at room temperature for 2 h. The following antibodies were used: EGFR (Santa Cruz Biotechnology), β-actin (Santa Cruz Biotechnology), p-p65(Servicebio, Wuhan, China), p-IκB (Servicebio, Wuhan, China), IKK (Servicebio, Wuhan, China), and secondary antibodies anti-mouse IgG-HRP (Millipore), anti-rabbit IgG-HRP (Millipore). Luminata Forte Western HRP substrate (Millipore) was used to visualize the bound antibodies.

### Cell Viability

cSCC cells HSC-1 and HSC-5 (4,000 per well) were seeded into 96-well plate and transfected with NC mimic or miR-27a mimic. CCK-8 (Yeasen, Shanghai, China) was added as described in the manual and OD values at 450 nm were detected after 2 h incubation.

### Cell Invasion Assay

Matrigel coated chambers (Corning) were used to assess the invasion ability of transfected cells. cSCC cells HSC-1 and HSC-5 (2.0 × 10^5^) transfected with NC mimic or miR-27a mimic were seeded into 8 μm chamber of 24-well plates in serum-free DMEM and the lower chambers were added with culture medium containing 10% FBS. After 16 h cultured at 37°C, the upper chambers were washed and fixed with fresh 3.7% formaldehyde. One hundred percent methanol were used to permeabilize cells, which were stained with 0.1% crystal violet and cell number analyzed by microphotograph.

### Luciferase Reporter Assay

The oligos containing the native or mutant binding site were cloned into pMIR-reporter vector (Promega). HEK293T cells were seeded into 12 well plates and co-transfected with pMIR-reporter constructs, renilla luciferase reporter vector, miR-27a mimic or NC mimic. Luciferase activities were measured at 48 h after transfection. The firely luciferase activity was normalized to renilla luciferase activity. The sequences of those oligos are listed in Supplementary Material.

### Subcutaneous Xenograft Model

BALB/c-nu/nu (male, 4–6 week old) were adopted from Guangdong Medical Laboratory Animal Center. The animal experiments were performed as described previously (15). HSC-5 or HSC-1 cells were transfected with NC mimic or miR-27a mimic. Cells (1.0 × 10^7^) were subcutaneously injected into the two flanks of nude mice. After 9 days of implantation, NC mimic or miR-27a mimic were injected into the respective tumors and repeated every 2 days. The tumor diameters were measured and recorded every day to generate a growth curve. The tumors were removed and feezed immediately for experiments followed. All procedures involving the mice were approved by the Southern Medical University Animal Care and Use Committee and in accordance with institutional guidelines.

### Statistical Analysis

The experimental results were represented with mean ± S.D. and Student's test or one-way ANOVA was used to analyze statistical difference. It was considered statistically significant when *P* < 0.05.

## Results

### miR-27a Is Sensitive to UVB Radiation in Epidermis

**Figure 1:**
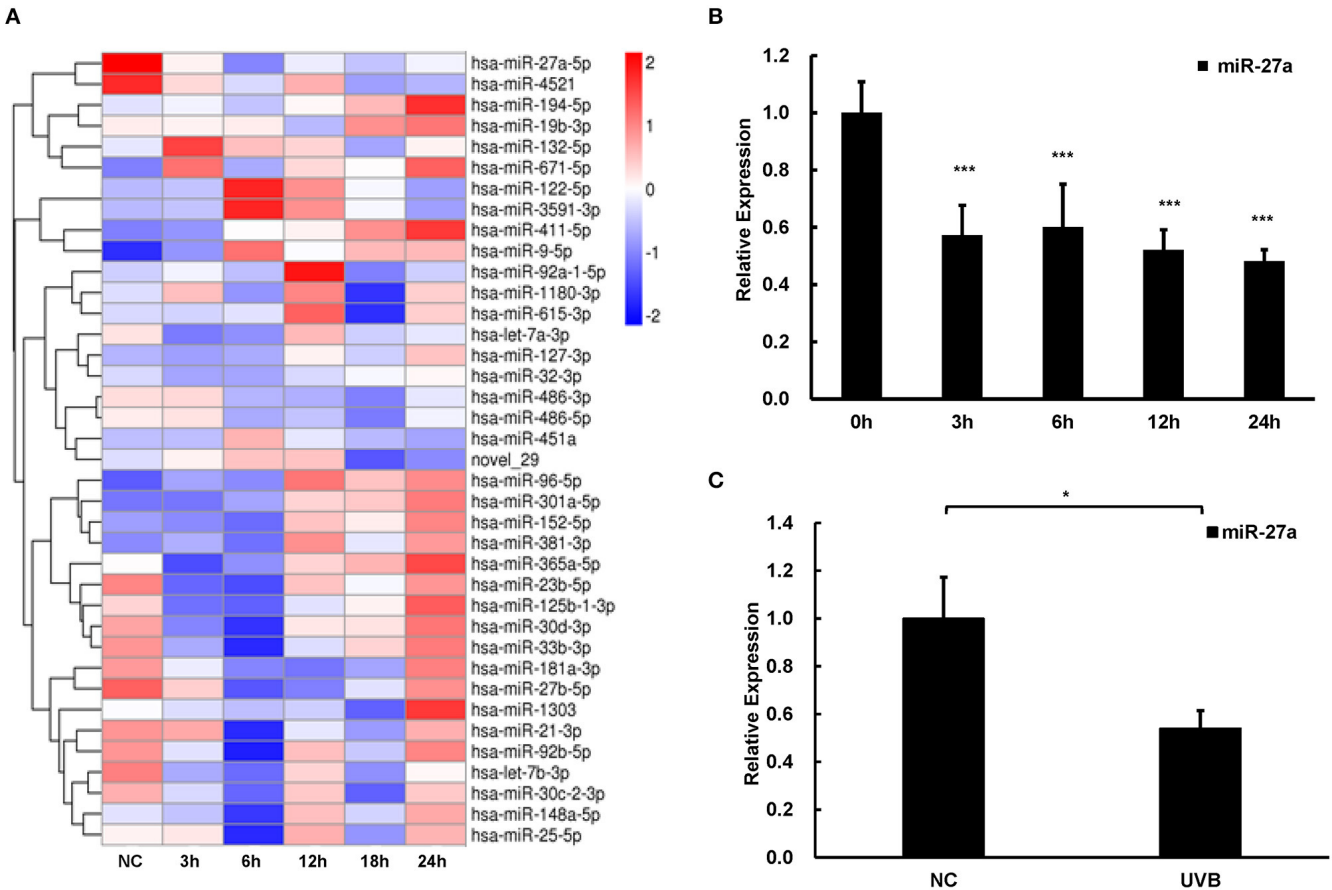
miR-27a was downregulated in respond to UVB radiation. **(A)** Hierarchical clustering analysis of differential expressed miRNAs at 3, 6, 12, 18, and 24 h after UVB radiation in HaCaT cells based on miRNA sequencing. **(B,C)** The expression of miR-27a was identified in HaCaT cells with UVB radiation by qPCR. Each experiment was performed in triplicate and data are presented as mean ± s.d. One-Way ANOVA and Dunnett's Multiple comparison test were used to analyze the data (*P < 0.05, ***P < 0.001).

UVB is the major pathogenic factor for cSCC. To discover miRNAs in response to UVB radiation and explore their functions in the progression of cSCC, we conducted miRNA sequencing to reveal those differentially expressed miRNAs in HaCaT cells at different time points (3, 6, 12, 18, and 24 h) after UVB radiation. Relative expression of miRNAs which were altered at least two-folds change at any time points compared with that in HaCaT cells without UVB radiation were selected and clustered using the hierarchical clustering algorithm (Figure 1A). miR-27a was differentially expressed in common at 6, 12, 18, and 24 h time points. The expression of miR-27a in HaCaT cells was significantly downregulated after UVB radiation verified by real-time PCR, which is coincident with RNA-seq (Figure 1B). In UVB-irradiated mice skin, miR-27a expression was significantly decreased compared with mice skins without UVB radiation (Figure 1C). Those results reminded us that miR-27a may play a vital role in the progression of cSCC.

### miR-27a Is Low Expressed in cSCC Cells and Tissues

**Figure 2:**
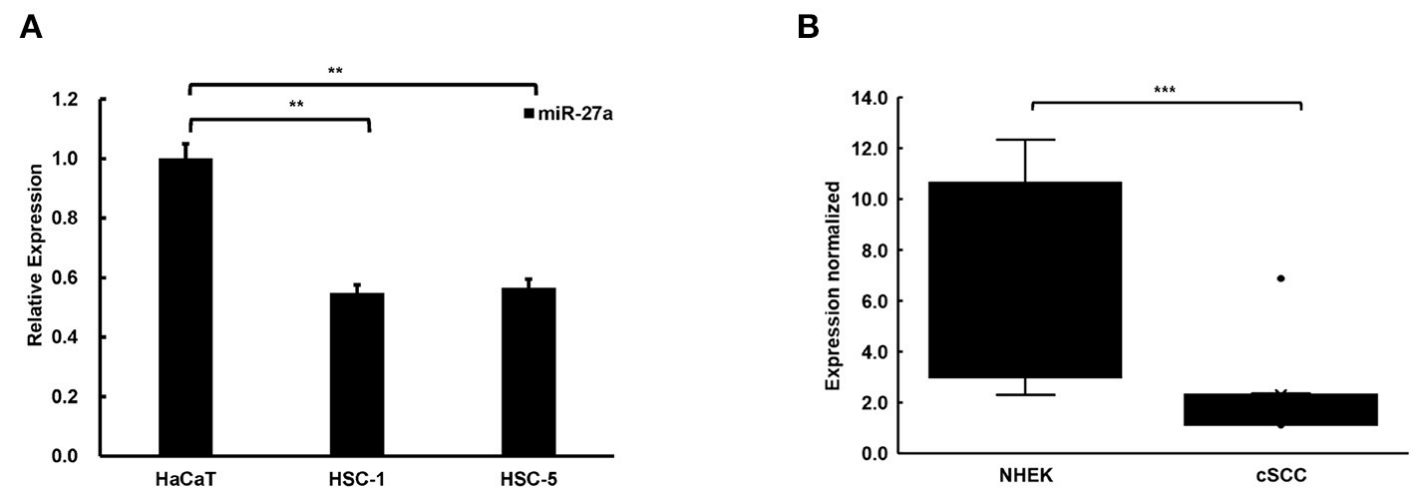
miR-27a was low expressed in cSCC. **(A)** The expression of miR-27a was downregulated in cSCC cells compared with HaCaT. **(B)** miR-27a expression was downregulated in cSCC tissues compared with NHEKs. Each experiment was performed in triplicate and data are presented as mean ± s.d. One-Way ANOVA and Dunnett's Multiple comparison test were used to analyze the data (**P<0.01, ***P < 0.001).

To explore the function of miR-27a in cSCC progression, we examined miR-27a expression in cSCC cell lines and tissues by qPCR. In contrast with HaCaT cells, miR-27a was dramatically low expressed in cSCC cells, HSC-5 and HSC-1 (Figure 2A). In the meantime, miR-27a expression was analyzed in cSCC patient tissues. Compared with normal human epidermal keratinocytes (NHEKs, *n* = 4), miR-27a was significantly reduced in cSCC tissues (*n* = 12) (Figure 2B). These results indicate that miR-27a is low expressed in cSCC.

### Loss of miR-27a Promotes Proliferation and Invasion of cSCC Cells

**Figure 3:**
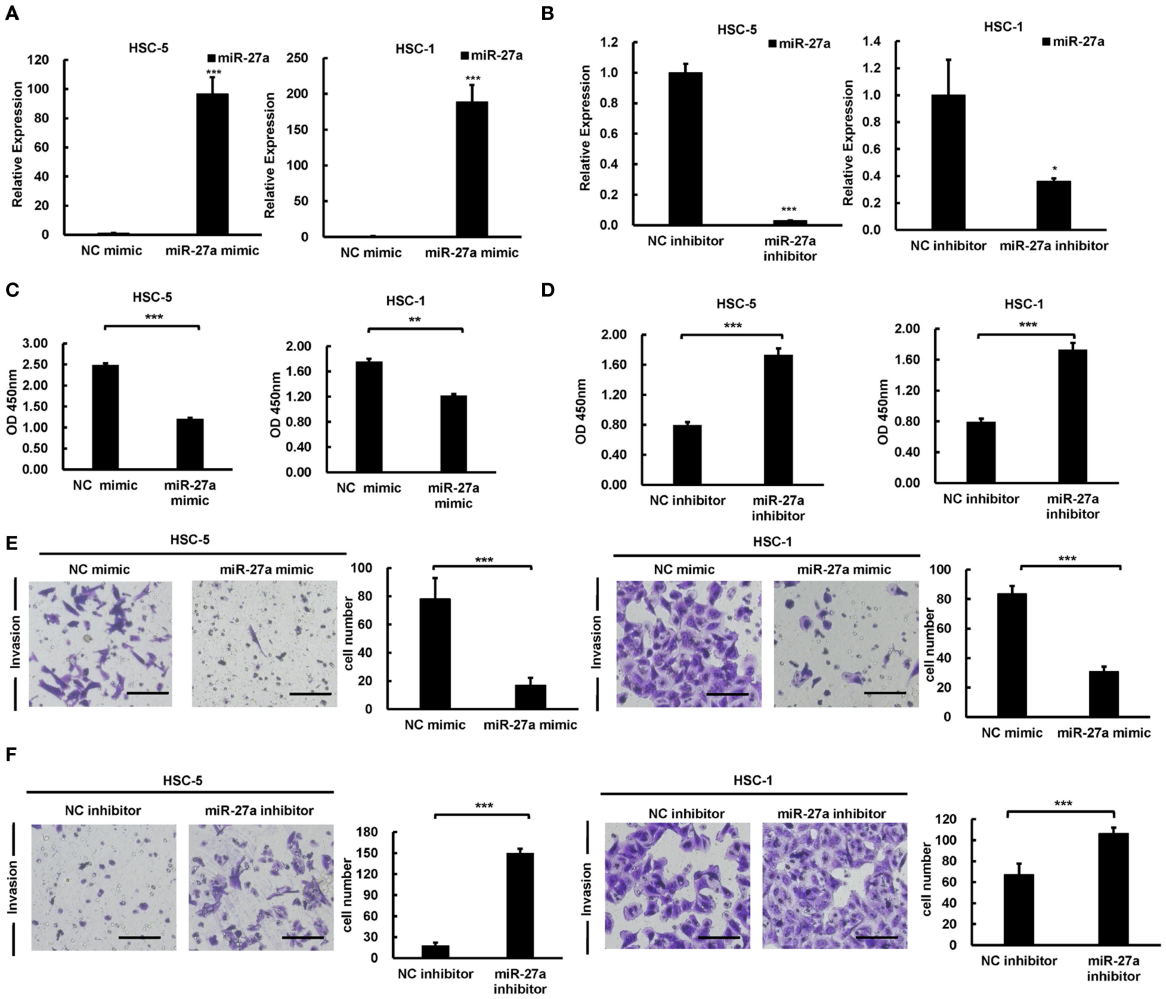
miR-27a acted as a tumor suppressive role in cSCC. Relative expression of miR-27a was detected in HSC-5 or HSC-1 cells treated with miR-27a mimic **(A)** or inhibitor **(B)**. Cell proliferation were determined by CCK-8 assays at 24, 48, and 72 h after transfection **(C,D)**. Cell invasions were detected by transwell assays with matrigels **(E,F)**. Magnification: 200×. Each experiment was performed in triplicate and data are presented as mean ± s.d. One-Way ANOVA and Dunnett's Multiple comparison test were used to analyze the data (*P < 0.05, **P < 0.01, ***P < 0.001).

To identify the effect of miR-27a in the development of cSCC, we regulated miR-27a expression in HSC-5 or HSC-1 cells by transfected miR-27a mimic or inhibitor. QPCR were performed to determined miR-27a expression. Result showed that miR-27 expression was significantly increased or downregulated in both HSC-5 or HSC-1 cells compared with negative control group (Figures 3A,B). Cell proliferation was determined by CCK-8 assays and the data showed proliferation inhibition by exogenous miR-27a (Figures 3C,D). Transwell assays were performed to detect the invasion of HSC-5 or HSC-1 cells with miR-27a mimic treatment. The results showed that the invasive ability of cSCC cells were suppressed after miR-27a mimic transfection (Figure 3E). Inversely, miR-27a inhibition in HSC-5 or HSC-1 cells were found to promote the invasive ability compared with cells transfected with negative control (Figure 3F). The data above demonstrated that miR-27a represses cell proliferation and invasiveness of cSCC cells *in vitro*.

### EGFR Is a Direct Target of miR-27a

**Figure 4:**
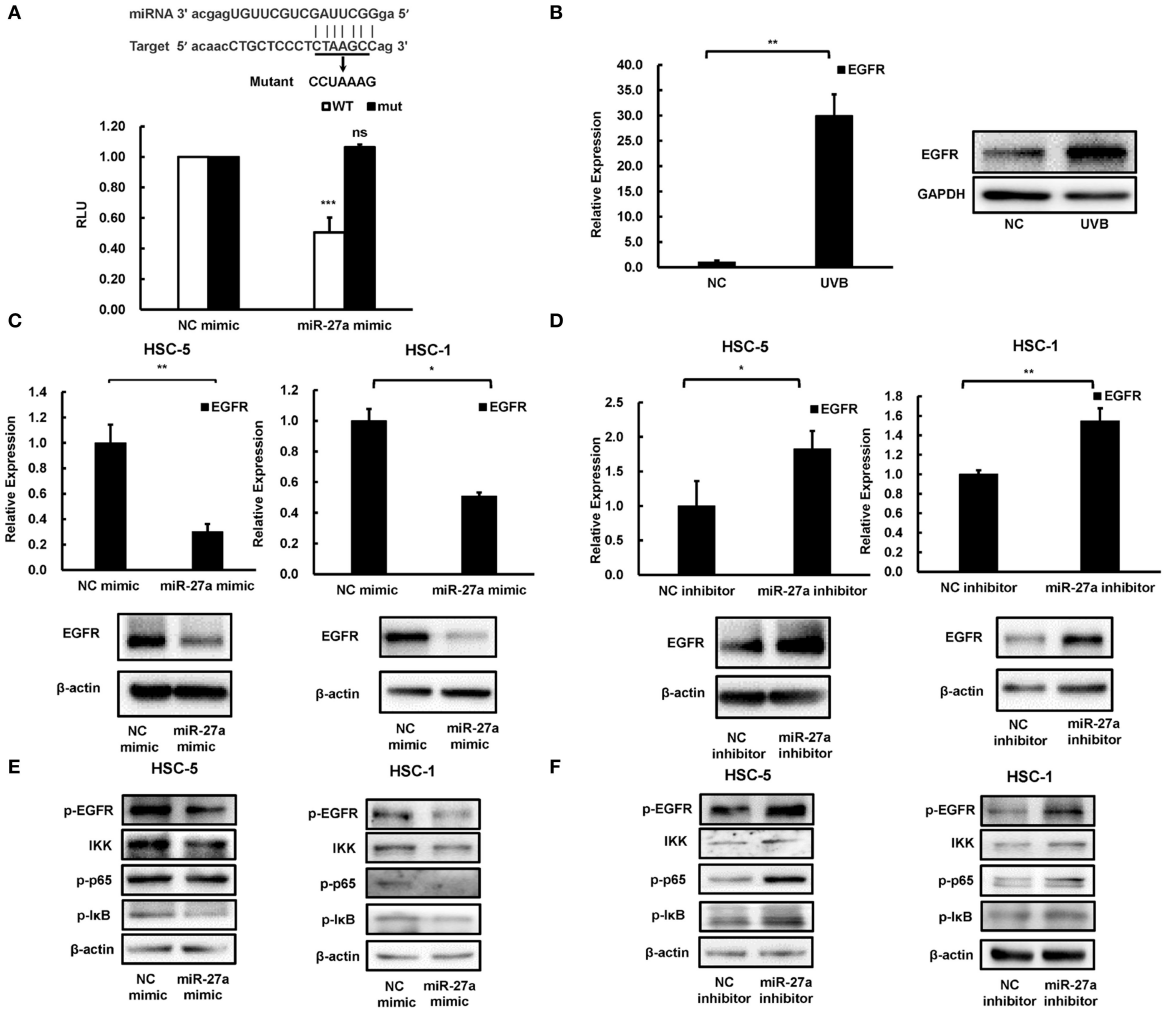
EGFR was a direct target of miR-27a. **(A)** miR-27a inhibits the wild type but not the mutant EGFR 3'UTR luciferase activity. **(B)** EGFR was induced in chronic UVB irradiated mice skins. **(C,D)** the expression of EGFR in cSCC cells transfected with miR-27a mimic or inhibitor were detected by qPCR and western blot. **(E,F)** miR-27a suppresses the phosphorylation of EGFR and regulates its downstream pathways. miR-27a overexpression or inhibition of miR-27a regulated the phosphorylation of EGFR and the phosphorylation of p65, IκB, and IKK. Each experiment was performed in triplicate. Each experiment was performed in triplicate and data are presented as mean ± s.d. One-Way ANOVA and Dunnett's Multiple comparison test were used to analyze the data (*P < 0.05, **P < 0.01, ***P < 0.001).

To explore the downstream of miR-27a, a bioinformatics screen was carried out by using miRTarBase (16). Consideration of the overactivation and crucial role of EGFR in most cancers, it was selected as the underlying downstream genes of miR-27a in cSCC cells. There was a binding site predicted for miR-27a in the 3'UTR region of EGFR mRNA (Figure 4A). Wild type or mutant binding site of EGFR were cloned and inserted into luciferase reporter vector. The luciferase reporter assays showed that miR-27a overexpression suppressed the luciferase activity of the wild type vector, while no significant change of the mutant vector (Figure 4A). In our previous study, EGFR was identified to be overexpressed in cSCC (14) and upregulated in mice skins receiving UVB radiation (Figure 4B), which was opposite to the expression of miR-27a. In HSC-5 or HSC-1 cells which were transfected with miR-27a mimic, EGFR were downregulated in both mRNA and protein level (Figure 4C); conversely, upregulated in miR-27a inhibitor treated HSC-5 or HSC-1 cells (Figure 4D). These results demonstrated that EGFR is a direct target of miR-27a in cSCC cells and miR-27a knockdown increases the expression of EGFR.

### miR-27a Modulates NF-κB Signaling Pathway via Inactivation of EGFR

To investigate the downstream mechanism of miR-27a targeting EGFR in cSCC cells, we detected the changes in the downstream signaling pathway after transfection of cSCC cells with miR-27a mimc or miR-27a inhibitor. Upregulation of miR-27a suppressed the activation of EGFR (Figure 4E) and the inhibition of miR-27a expression increased the phosphorylation of EGFR (Figure 4F). Therefore, we further investigated whether the downstream pathways of EGFR were modulated by miR-27a. Results showed that miR-27a inhibited the phosphorylation of p65, IKK, and IκB while downregulation of miR-27a increased the phosphorylation of p65, IKK, and IκB, which indicates miR-27a suppresses the activation of NF-κB pathway (Figures 4E,F). These results suggested that miR-27a inhibits cSCC development by targeting EGFR and its downstream NF-κB signaling pathway.

### miR-27a Inhibits the Growth of cSCC *in vivo*

**Figure 5:**
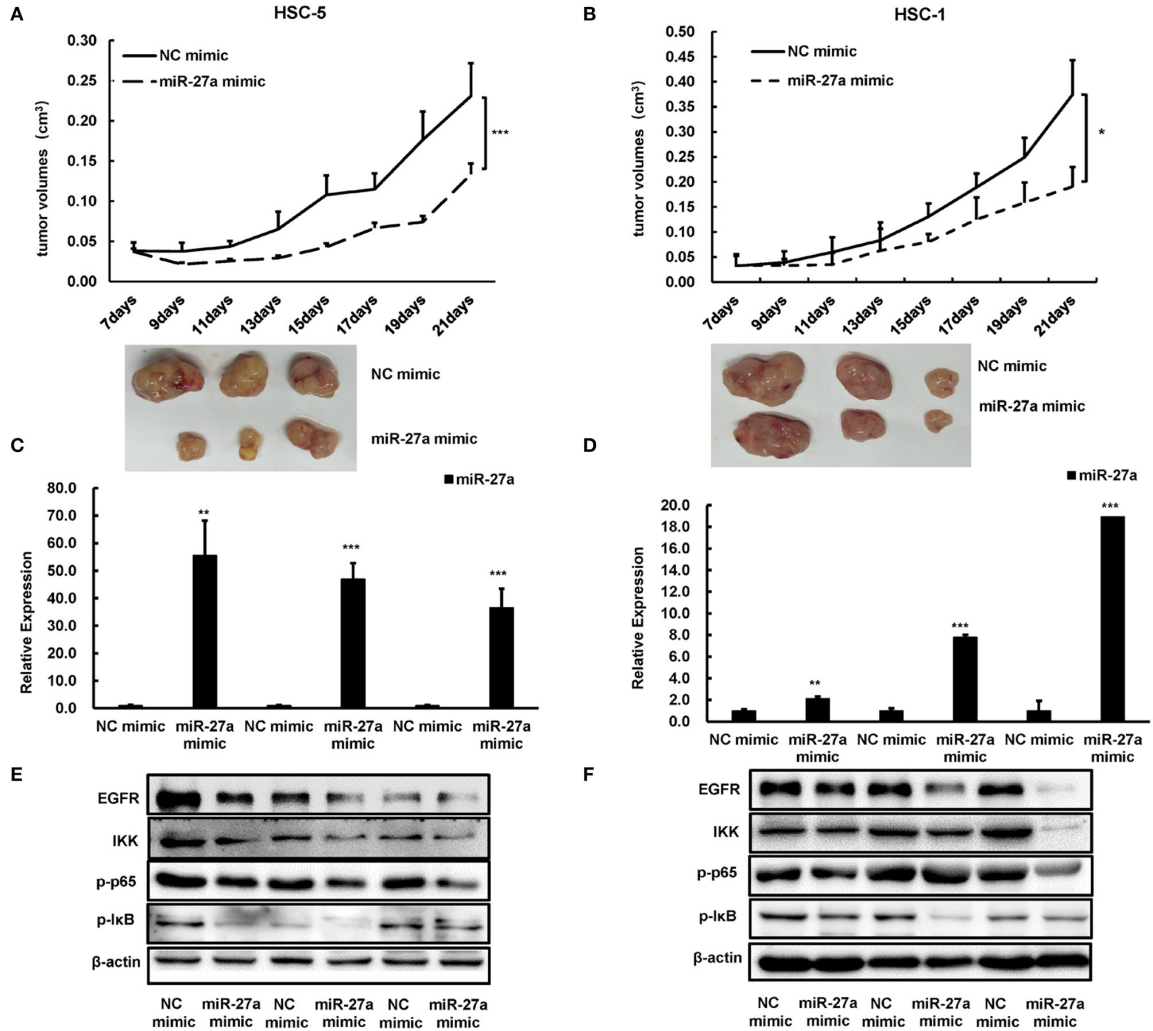
miR-27a inhibited tumor growth of cSCC *in vivo*. **(A,B)** Growth curves of tumor volumes in miR-27a mimic group and NC mimic group were determined every 3 days. Representative photographs of tumors were shown below. **(C,D)** Relative expression of miR-27a in HSC-5 cells treated with miR-27a mimic or negative control. **(E,F)** miR-27a overexpression regulated the phosphorylation of EGFR and the phosphorylation of p65, IκB, and IKK. Each experiment was performed in triplicate and data are presented as mean ± s.d. One-Way ANOVA and Dunnett's Multiple comparison test were used to analyze the data (*P < 0.05, **P<0.01, ***P <0.001).

To evaluate the antitumor function of miR-27a *in vivo*, we established a subcutaneous model. miR-27a mimic or NC mimic was delivered into HSC-5 or HSC-1 cells. The tumor volume was significantly decreased in the group treated with miR-27a mimic compared with that in the negative control group (Figures 5A,B). The tumors were removed after implantation for 21 days and miR-27a expression in the xenografts were determined by qPCR. miR-27a was upregulated in the xenografts treated with miR-27a mimic compared the negative control group (Figures 5C,D). Furthermore, miR-27a led to the significant downregulation of EGFR and its downstream NF-κB signaling pathway (Figures 5E,F), which is consistent with that *in vitro*.

## Discussion

UV is a major environmental carcinogenesis for the initiation and promotion of cSCC. It has been estimated that about 93% of skin cancers could been due to UV exposure. Accumulating data show that miRNAs play a vital role in modulating cell proliferation and invasion by regulating their target genes. In previous studies, we screened and verified miRNAs that are upregulated by UVB radiation in HaCaT cells play an onco-miR role in cSCC (8, 10). However, the role of miR-27a in cSCC remains to be elucidated. It is the first time uncovering the function of UVB-sensitive miR-27a in the development of cSCC. In this study, we revealed that miR-27a inhibits cell proliferation and invasion of cSCC, and suppresses the activation of NF-κB pathway through directly targeting EGFR, indicating that miR-27a plays a tumor suppressive role in cSCC.

miR-27a abnormal expression and mutation has been identified in various malignant tumors, such as breast, renal and colorectal cancer (13, 17, 18). miR-27a decreased the risk of breast cancer in Caucasians (17) and population with the genetic variant of pre-miR-27a had a lower risk of renal cell carcinoma (RCC) (13). Further, low expressed miR-27a was related to high grade in colorectal cancer (18). miR-27a inhibited A549 cell proliferation via MET signaling (19) and in esophageal squamous cell carcinoma functioned as a tumor suppressor through binding to oncogene KRAS (20). Consistent with these studies, we found declined levels of miR-27a in cSCC and exogenous miR-27a suppresses cell proliferation and leads to the metastasis inhibition of cSCC significantly *in vivo* and *in vitro*.

EGFR is considered playing a crucial role in regulating cellular processes, including cell proliferation, differentiation and migration during development and homoeostasis (21). In most cancers, especially in most epithelial tumors, EGFR is commonly upregulated and closely associated with poor differentiation or unfavorable prognosis (22–24). Previous studies show that overexpression or constitutive activation of EGFR contributes to cell survival, proliferation, and invasiveness in cSCC (14, 22). In this study, we identified EGFR as a direct target gene of miR-27a by luciferase reporter assay. Exogenous transfected miR-27a led to a decreased level of EGFR and inhibition of miR-27a led to EGFR increased in cSCC cells, indicating that miR-27a suppressed the expression of EGFR by binding to 3′UTR region, which is consistent with the tumor suppressive role of miR-27a in cSCC. As it is getting great attention for EGFR targeted therapies, such as EGFR inhibitors, some clinical trials targeting EGFR have been conducted aiming at cSCC therapy (25, 26). However, it still needs to discover new strategies for cSCC treatment due to the toxicity of EGFR inhibitors and resistance to EGFR (27). NF-κB is highly conserved transcription factor and NF-κB complexes are localized in the cytoplasm in inactivated state (28). In the canonical pathway, IKK is activated and phosphorylates IκB. IκB is subsequently ubiquitinated to release p65, which lead to its nuclear translocation (29). EGFR signals triggers NF-κB activation through IKK complex and the phosphorylation of IκB (30) which is abnormally constitutively activated in cancer cells and driving tumorigenesis by promoting cell proliferation and metastasis (31, 32). In addition, NF-κB activation also promotes the resistance to EGFR inhibitors, which leads to a reduced therapeutic effectiveness of EGFR inhibitors (33). Consistent with these studies, our data showed that miR-27a downregulates EGFR expression by directly binding to the 3′UTR region. Further, miR-27a inhibits the phosphorylation of EGFR and its downstream NF-κB pathway, which repress the phosphorylation of IKK and IκB to inhibit the function of IKK complex and inactivation of NF-κB activation.

**Figure 6:**
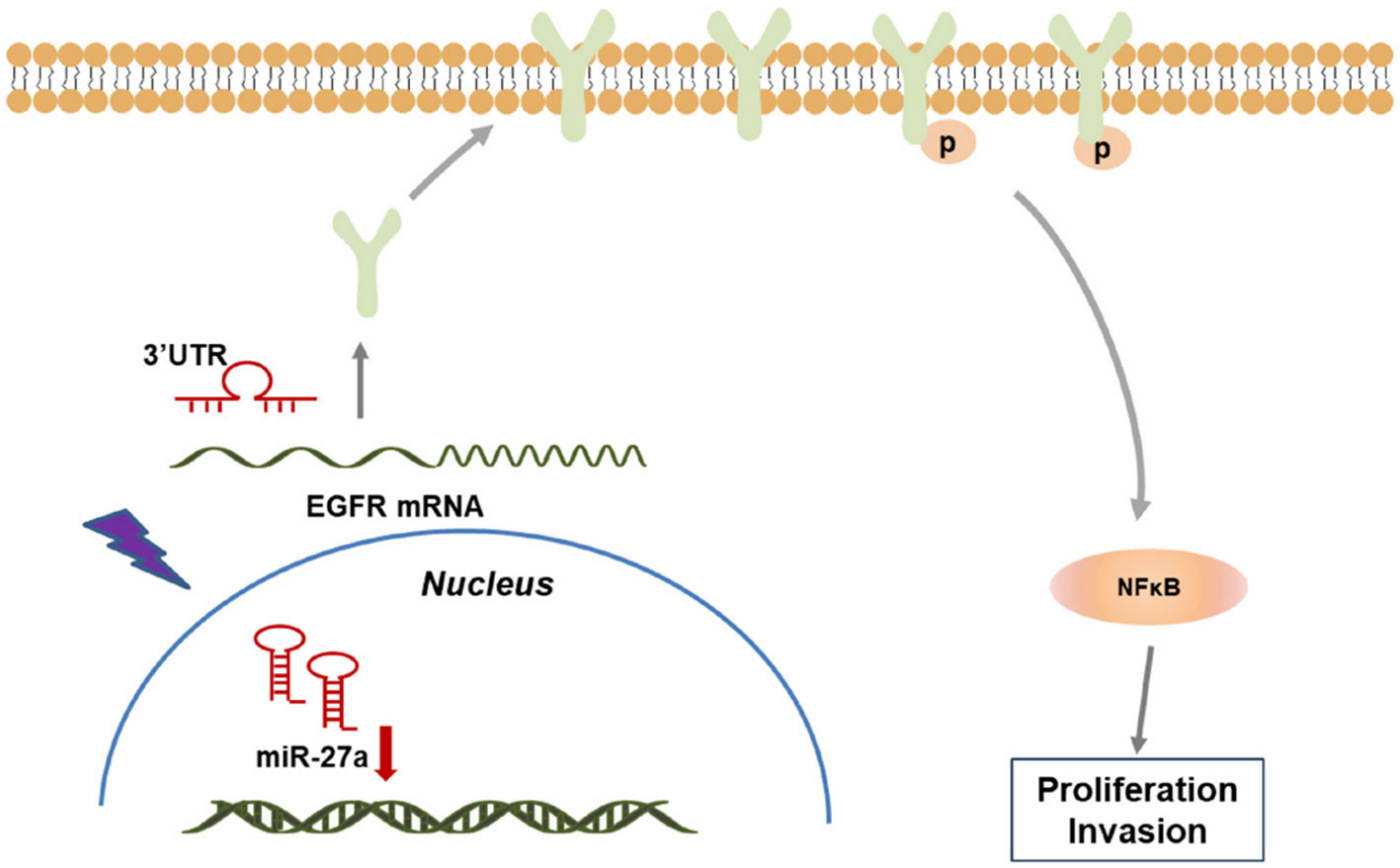
A model of miR-27a-EGFR- NF-κB regulatory axis in cSCC development. In cSCC tumors, loss of miR-27a up-regulates EGFR and the downstream NF-κB pathway, which contributes to the enhancement of proliferation and metastasis, thus promotes cSCC progression.

Together, our study demonstrated that miR-27a inhibits the progression of cSCC via targeting EGFR and its downstream NF-κB pathway. miR-27a was selected as a sensitivity miRNA in response to UVB radiation and downregulated in cSCC. miR-27a inhibited the proliferation and metastasis of cSCC cells. Furthermore, EGFR was identified to be directly targeted by miR-27a, which restrained the activation of NF-κB via directly targeting EGFR, which indicates that miR-27a may act as a tumor suppressor through NF-κB pathway (Figure 6). Our findings reveal a regulatory axis of miR-27a-EGFR-NF-κB that may be a novel putative therapeutic target for cSCC.

## Data Availability Statement

The datasets generated for this study are available on request to the corresponding author.

## Ethics Statement

The studies involving human participants were reviewed and approved by Institutional Review Board of Nanfang Hospital affiliated to Southern Medical University. The patients/participants provided their written informed consent to participate in this study. The animal study was reviewed and approved by The Southern Medical University Animal Care and Use Committee. Written informed consent was obtained from the individual(s) for the publication of any potentially identifiable images or data included in this article.

## Author Contributions

MZ and YW conceived and designed the experiments. YW, XD, XN, and YD performed the experiments. YW and XD analyzed the data. MZ, YW, and XD wrote the paper, reviewed, and edited the manuscript.

### Conflict of Interest

The authors declare that the research was conducted in the absence of any commercial or financial relationships that could be construed as a potential conflict of interest.

## References

[1] Que SKT Zwald FO Schmults CD. Cutaneous squamous cell carcinoma: incidence, risk factors, diagnosis, and staging. J Am Acad Dermatol. (2018) 78:237–47. 10.1016/j.jaad.2017.08.059 29332704

[2] Nehal KS Bichakjian CK. Update on Keratinocyte Carcinomas. N Engl J Med. (2018) 379:363–74. 10.1056/NEJMra1708701 30044931

[3] Bowden GT. Prevention of non-melanoma skin cancer by targeting ultraviolet-B-light signalling. Nat Rev Cancer. (2004) 4:23–35. 10.1038/nrc1253 14681688

[4] Esteller M. Non-coding RNAs in human disease. Nat Rev Genet. (2011) 12:861–74. 10.1038/nrg3074 22094949

[5] Smith B Agarwal P Bhowmick NA. MicroRNA applications for prostate, ovarian and breast cancer in the era of precision medicine. Endocr Relat Cancer. (2017) 24:R157–72. 10.1530/ERC-16-0525 28289080 PMC5446589

[6] Wu SG Chang TH Liu YN Shih JY MicroRNA in lung cancer metastasis. Cancers. (2019) 11:E265 10.3390/cancers11020265 30813457 PMC6406837

[7] Shah MY Ferrajoli A Sood AK Lopez-Berestein G Calin GA. microRNA therapeutics in cancer - an emerging concept. EBioMedicine. (2016) 12:34–42. 10.1016/j.ebiom.2016.09.017 27720213 PMC5078622

[8] Konicke K Lopez-Luna A Munoz-Carrillo JL Servin-Gonzalez LS Flores-de la Torre A Olasz E. The microRNA landscape of cutaneous squamous cell carcinoma. Drug Discov Today. (2018) 23:864–70. 10.1016/j.drudis.2018.01.023 29317340

[9] Li X Zhou C Zhang C Xie X Zhou Z Zhou M. MicroRNA-664 functions as an oncogene in cutaneous squamous cell carcinomas (cSCC) via suppressing interferon regulatory factor 2. J Dermatol Sci. (2019) 94:330–8. 10.1016/j.jdermsci.2019.05.004 31138473

[10] Zhou M Liu W Ma S Cao H Peng X Guo L. A novel onco-miR-365 induces cutaneous squamous cell carcinoma. Carcinogenesis. (2013) 34:1653–9. 10.1093/carcin/bgt097 23514750 PMC3697891

[11] Vimalraj S Miranda PJ Ramyakrishna B Selvamurugan N. Regulation of breast cancer and bone metastasis by microRNAs. Dis Markers. (2013) 35:369–87. 10.1155/2013/451248 24191129 PMC3809754

[12] Ye P Ke X Zang X Sun H Dong Z Lin J. Up-regulated miR-27-3p promotes the G1-S phase transition by targeting inhibitor of growth family member 5 in osteosarcoma. Biomed Pharmacother. (2018) 101:219–27. 10.1016/j.biopha.2018.02.066 29494959

[13] Shi D Li P Ma L Zhong D Chu H Yan F. A genetic variant in pre-miR-27a is associated with a reduced renal cell cancer risk in a Chinese population. PLoS ONE. (2012) 7:e46566. 10.1371/journal.pone.0046566 23118855 PMC3484143

[14] Zhang Y Gao L Ma S Ma J Wang Y Li S. MALAT1-KTN1-EGFR regulatory axis promotes the development of cutaneous squamous cell carcinoma. Cell Death Differ. (2019) 26:2061–73. 10.1038/s41418-019-0288-7 30683916 PMC6748142

[15] Zhou L Wang Y Zhou M Zhang Y Wang P Li X. HOXA9 inhibits HIF-1alpha-mediated glycolysis through interacting with CRIP2 to repress cutaneous squamous cell carcinoma development. Nat Commun. (2018) 9:1480. 10.1038/s41467-018-03914-5 29662084 PMC5902613

[16] Chou CH Shrestha S Yang CD Chang NW Lin YL Liao KW. miRTarBase update 2018: a resource for experimentally validated microRNA-target interactions. Nucleic Acids Res. (2018) 46:D296–302. 10.1093/nar/gkx1067 29126174 PMC5753222

[17] Zhang H Zhang Y Zhao X Ma X Yan W Wang W. Association of two microRNA polymorphisms miR-27 rs895819 and miR-423 rs6505162 with the risk of cancer. Oncotarget. (2017) 8:46969–80. 10.18632/oncotarget.16443 28415619 PMC5564537

[18] Bao Y Chen Z Guo Y Feng Y Li Z Han W. Tumor suppressor microRNA-27a in colorectal carcinogenesis and progression by targeting SGPP1 and Smad2. PLoS ONE. (2014) 9:e105991. 10.1371/journal.pone.0105991 25166914 PMC4148394

[19] Yang Y Zang A Jia Y Shang Y Zhang Z Ge K. Genistein inhibits A549 human lung cancer cell proliferation via miR-27a and MET signaling. Oncol Lett. (2016) 12:2189–93. 10.3892/ol.2016.4817 27602162 PMC4998592

[20] Zhu L Wang Z Fan Q Wang R Sun Y. microRNA-27a functions as a tumor suppressor in esophageal squamous cell carcinoma by targeting KRAS. Oncol Rep. (2014) 31:280–6. 10.3892/or.2013.2807 24154848

[21] Sigismund S Avanzato D Lanzetti L. Emerging functions of the EGFR in cancer. Mol Oncol. (2018) 12:3–20. 10.1002/1878-0261.12155 29124875 PMC5748484

[22] Canueto J Cardenoso E Garcia JL Santos-Briz A Castellanos-Martin A Fernandez-Lopez E. Epidermal growth factor receptor expression is associated with poor outcome in cutaneous squamous cell carcinoma. Br J Dermatol. (2017) 176:1279–87. 10.1111/bjd.14936 27510450

[23] Wee P Wang Z. Epidermal growth factor receptor cell proliferation signaling pathways. Cancers. (2017) 9: E52. 10.3390/cancers9050052 28513565 PMC5447962

[24] Sabattini S Marconato L Zoff A Morini M Scarpa F Capitani O. Epidermal growth factor receptor expression is predictive of poor prognosis in feline cutaneous squamous cell carcinoma. J Feline Med Surg. (2010) 12:760–8. 10.1016/j.jfms.2010.04.010 20674427 PMC11135530

[25] William WN Jr Feng L Ferrarotto R Ginsberg L Kies M Lippman S. Gefitinib for patients with incurable cutaneous squamous cell carcinoma: A single-arm phase II clinical trial. J Am Acad Dermatol. (2017) 77:1110–3.e2. 10.1016/j.jaad.2017.07.048 28964539 PMC5685879

[26] Lewis CM Glisson BS Feng L Wan F Tang X Wistuba II. A phase II study of gefitinib for aggressive cutaneous squamous cell carcinoma of the head and neck. Clin Cancer Res. (2012) 18:1435–46. 10.1158/1078-0432.CCR-11-1951 22261807 PMC6167936

[27] Shostak K Chariot A. EGFR and NF-kappaB: partners in cancer. Trends Mol Med. (2015) 21:385–93. 10.1016/j.molmed.2015.04.001 25979753

[28] May MJ Ghosh S. Rel/NF-kappa B and I kappa B proteins: an overview. Semin Cancer Biol. (1997) 8:63–73. 10.1006/scbi.1997.0057 9299584

[29] Hayden MS Ghosh S. Shared principles in NF-kappaB signaling. Cell. (2008) 132:344–62. 10.1016/j.cell.2008.01.020 18267068

[30] Le Page C Koumakpayi IH Lessard L Mes-Masson AM Saad F. EGFR and Her-2 regulate the constitutive activation of NF-kappaB in PC-3 prostate cancer cells. Prostate. (2005) 65:130–40. 10.1002/pros.20234 15880609

[31] Dolcet X Llobet D Pallares J Matias-Guiu X. NF-kB in development and progression of human cancer. Virchows Arch. (2005) 446:475–82. 10.1007/s00428-005-1264-9 15856292

[32] Barkett M Gilmore TD. Control of apoptosis by Rel/NF-kappaB transcription factors. Oncogene. (1999) 18:6910–24. 10.1038/sj.onc.1203238 10602466

[33] Kobayashi S Boggon TJ Dayaram T Janne PA Kocher O Meyerson M. EGFR mutation and resistance of non-small-cell lung cancer to gefitinib. N Engl J Med. (2005) 352:786–92. 10.1056/NEJMoa044238 15728811

